# Patients with Dipper and Nondipper High-Normal Blood Pressure Were Associated with Left Ventricular Mass

**DOI:** 10.1155/2021/6946418

**Published:** 2021-12-21

**Authors:** Fan-kai Xiao, Ping Li, Zhan-ying Han, Li Jing, Shaohua Hua, Luo-sha Zhao

**Affiliations:** ^1^Oncology Department, The First Affiliated Hospital of Zhengzhou University, Zhengzhou, China; ^2^Department of Cardiology and Hypertension, First Affiliated Hospital of Zhengzhou University, Zhengzhou, China; ^3^Henan Medical College, Zhengzhou, China; ^4^Department of Ultrasound, The First Affiliated Hospital of Zhengzhou University, Zhengzhou, China

## Abstract

**Purpose:**

High-normal blood pressure has been suggested to associate with target organ damage and higher left ventricular mass index (LVMI). Our aim is to find the association between people with high-normal blood pressure and their left ventricular mass index.

**Materials and Methods:**

Given a total of 181 people with office blood pressure, 24-hour ambulatory blood pressure monitoring, 35 of them are normotensive (BP < 130/85 mm Hg), and 146 people with high-normal blood pressure (BP 130–139/85–89 mm Hg), divide the high-normal blood pressure group into dipper and nondipper according to their ABPM in 24 hours. All of them were performed with echocardiography to calculate LVMI.

**Results:**

After adjusting for potential confounding factors, mean systolic blood pressure (BP) of the nondipper group is (119 + 9) mmHg in 24 h, which is significantly higher (*p* < 0.05) than in the dipper group (116 + 11) mmHg, indicating the mean systolic BP is associated with the dipper type (*p* < 0.05); furthermore, the higher nocturnal blood pressure is associated with the nondipper group significantly (*p* < 0.05), and LVMI ((121 ± 11) g/m^2^) of the nondipper group is also significantly higher than in the dipper group's LVMI ((108 ± 12) g/m^2^) (*p* < 0.05). The multivariate linear regression analyses revealed significant and independent associations of LVMI with these factors: triglyceride (TG), total cholesterol (TC), low-density lipoprotein (LDL-C), and coefficient of variation of systolic and diastolic blood pressure in 24 hours.

**Conclusion:**

After multiple relevant clinical confounding factors were adjusted, patients with dipper and nondipper high-normal blood pressure had higher LVMI. Abnormalities in circadian blood pressure variability may be associated with the left ventricular hypertrophy.

## 1. Introduction

According to the 2020 international hypertension guideline, high-normal blood pressure was defined as systolic BP between 130 and 139 mmHg and/or diastolic BP between 85 and 89 mmHg based on 2 or more properly measured seated BP readings on each of 2 or more office visits [1]. Report from Yuli indicated that high-normal blood pressure has increased the risk of cardiovascular disease after adjusting for multiple factors even in low range; meanwhile, it is also harmful to end-stage renal disease or chronic kidney disease, fundus lesion, and endothelia cell abnormality [2].

Recently, although, 24-hour ambulatory blood pressure monitoring (ABPM) was recommended to apply for the prevention, evaluation, detection, and treatment of both high-normal blood pressure and hypertension in patients [3]. There is less investigation on the impact of high-normal BP on LV mechanics or left ventricular hypertrophy through ABPM.

We aimed to find the relation between LVMI and high-normal blood pressure in both dipper and nondipper through analysis of high-normal blood pressure and normotensive by ABPM.

### 1.1. Patients and Methods

Recruitment: 146 consecutive patients who were diagnosed to high-normal blood pressure and 35 normotensive people who took general cardiovascular check-up from the cardiology outpatient clinic in the First Affiliated Hospital of Zhengzhou University were involved. According to the 2020 ISH Global Hypertension Practice Guidelines, based on expert opinion, panel recommends systolic BP between 130 and 139 mmHg and/or diastolic BP between 85 and 89 mmHg [1]. We choose normotensive groups aged from 18 to 50 years old, systolic pressure under 130 mmHg, and diastolic blood pressure under 85 mmHg. The standard of high-normal blood pressure followed 2020 ISH Global Hypertension Practice Guidelines, those with SBP 130–139 mmHg or DSB 85–89 mmHg. Exclusive criteria were diabetes, chronic kidney disease, coronary artery disease, peripheral artery disease, heart failure, previous stroke, and unwilling to provide written informed consent. 181 people (146 high-normal blood pressure and 35 normotensive) who were eligible to the above condition and exclusive criteria were enrolled. Then, according to the results of ABPM, the high-normal blood pressure group could be divided into the dipper group (*n* = 77) and nondipper group (*n* = 69).

Procedure: recorded the age, sex, height, body mass index (BMI), heart rate, blood pressure, fasting blood glucose (FBG) lipid, liver function test, renal function test, routine blood test, whether smoke, whether have hypertension family history or not, and electrocardiograph (ECG).

BP measurements were taken in the sitting position after 5 minutes of resting using nondominant arm kept in the same line with the heart. DBP and SBP were calculated twice in different days, and record the average of them.

The FDA and CE certified ambulatory blood pressure monitoring (ABPM) (CONTEC, China) [4, 5] (https://contecmedical.en.made-in-china.com/product/TSLEawYPqgpk/China-Contec-Abpm50-Abpm-Bp-Holter-Ambulatory-Blood-Pressure-Monitor-CE-and-FDA.html) was performed using the noninvasive CB-1805-B recorder. Day time was defined as 6:00 am–22:00 pm, BP recordings were taken every 20 minutes, and night time was defined as 22:00 pm–6:00 am, BP recordings were taken every 30 minutes. According to the formula of the percentage of nocturnal BP fall, 100 × [1-(average night SBP/average day SBP)], patients were defined as dippers if their nocturnal BP fall was equal or greater than 10%, overdippers if greater than 20%, and nondippers if 0%–10%. The reverse dippers were regarded less than 0%. As supplementary, this research had found no overdippers and reverse dippers.

**Figure 1:**
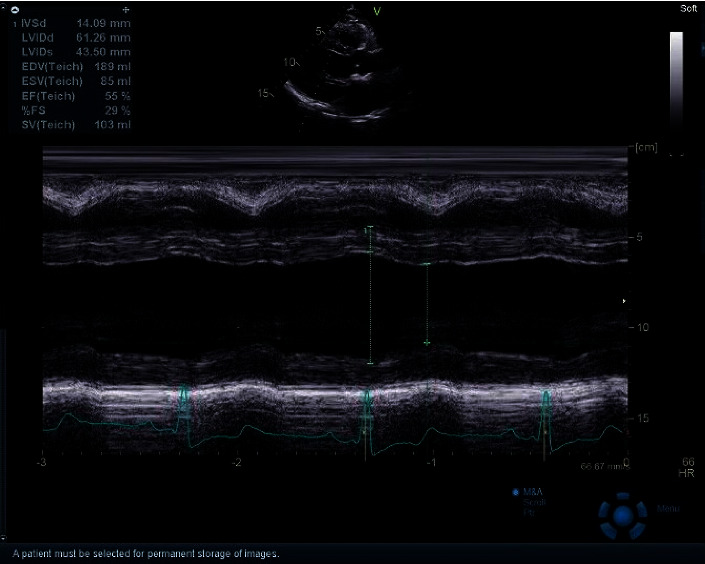
Echocardiographic analysis of left ventricular mass.

Echocardiographic measurements were performed in the left lying position, using GE vivid E95 ultrasound with a 2.0–4.0 MHz transducer (Figure 1). Left atrial diameter (LA), interventricular septum diameter (IVSD), left ventricular end diastolic diameter (EDD), and posterior wall thickness in diastole (PWTD) were acquired according to the standard operation of the echocardiography. LVMI was calculated through the formula of Devereux.

The SPSS 21 was applied for all statistical calculation. Continuous variables were defined as mean ± standard deviation (SD) and were compared by analysis of variance. Normal distribution was assessed through the Shapiro–Wilk test. The categorical variables between two groups were obtained through the *χ*^2^ test and were showed as percentages. Statistical significance was defined as *p* < 0.05.

## 2. Results

Our results were adjusted for major potential confounders, including disease history and sex, as an integral part of the analysis. Dipper, nondipper, and normotensive show no significant difference (*p* > 0.05) in ages, sex, FBG, high-density lipoprotein cholesterol (HDL-C), and low-density lipoprotein cholesterol (LDLC). Dipper and nondipper were significantly higher than the normotensive in percentage of the family history of hypertension, BMI, and cholesterol. Furthermore, the nondipper group has significantly higher TG than both dipper and normotensive groups (*p* < 0.05) (Table 1).

Average 24 h blood pressure in different types of SBP and DSB of both dipper and nondipper groups is higher than in the normotensive group. Coefficient variation of 24 h SBP of nondipper is higher than normotensive (Table 2).

LVMI was higher in high-normal blood pressure patients compared with the normotensive (nondipper was 121 ± 11 g/m^2^, dipper was 108 ± 13 g/m^2^, and normotensive was 98 ± 17 g/m^2^); furthermore, nondipper was significantly higher than dipper (*p* < 0.05).

Pearson's correlation coefficients for the association between LVMI and SBP, DBP, circadian rhythm of blood pressure, age, BMI,TG, TC, HDL-C, LDL-C, coefficient variation of 24 h SBP, and coefficient variation of 24 h DBP are given in Table 3.

## 3. Discussion

According to the research of Framingham heart study, higher BP will increase the risk of cardiovascular disease, and it was reported that malignant circadian rhythm of blood pressure has a close relationship with the damage of cardiovascular because it was meaningful to judge the damage and prognosis of target organ induced by high-normal blood pressure [6].

Although there are many research studies in the risk factors of hypertension in patients with higher LVMI and left ventricular hypertrophy, studies on the relationship between high-normal blood pressure and LVMI are limited. Previous studies [7, 8] reported that the major type of left ventricular hypertrophy is more popular in nondippers through investigation in patients with treated hypertension, and one study indicated that LVMI is higher in high-normal blood pressure than normotensive [9]. Ekrem et al. reported that patients with prehypertension belonging to nondippers have more impaired LV diastolic function than patients with prehypertension of dipper [10]. Maybe this is due to the fact that only subjects who had a clinical indication to perform ABPM were enrolled in the study. Furthermore, prehypertension includes BP values of 120–139/80–89 mmHg, whereas high-normal BP includes BP values of 130–139/85–89 mmHg. In addition, high-normal blood pressure is probably associated with detrimental alterations of the left ventricle. Cuspidi showed that persistent high-normal blood pressure was correlated with a higher risk of concentric remodeling, left ventricle hypertrophy, and a poor diastolic function [11, 12]. Similarly, after adjustment for various confounding factors, our results demonstrated that LVMI is higher in high-normal blood pressure than normotensive, and LVMI is increased in the nondipper group compared to the dipper group.

To the high-normal blood pressure patients, recent studies recommended that it is more efficient and reliable to monitor their blood pressure through ABPM [13, 14]. Through the 24 hours ABPM, our study found a significant difference in the increase of blood pressure between normotensive and high-normal blood pressure, especially in systolic blood pressure. Another retrospective study proved that higher DSBP, DDBP, NSBP, and NDBP will increase the risk of ischemic cerebrovascular and cardiovascular events [15]. Our research indicated that 24 h SSD was significantly higher in the high-normal blood pressure group than normotensives (*p* < 0.01); what is more, coefficient of the variation value is also higher in the high-normal blood pressure group. According to the study of Parati [16], the higher of the BPV, the greater damage of the heart or other target organs would be taken. The present study confirms that it is easier for the high-normal blood pressure group to take target organs damage compared to the normotensive, and left ventricular is one of the damaged target organs. Although the mechanism is not clearly understood, many studies proposed that higher blood pressure would increase the tension of vascular smooth muscle and induced hypertrophy and hyperplasia of vascular smooth muscle cells, impaired large arteries elasticity, and increased aortic stiffness; meanwhile, pulse pressure would increase and cause the tension of vascular smooth muscle further raise. This vicious circle would induce atherosclerosis and damage target organs [17, 18].

Abnormalities in circadian blood pressure variability is one of the most important reasons that induces higher LVMI. Recent studies indicated that the high-normal blood pressure patients have abnormalities in circadian blood pressure variability due to the flowing reasons: first, autonomic nervous system function was dysregulation in the early stage of hypertension; hyperfunction of vagus nerve and imbalance of function of parasympathetic nervous during midnight will increase the nocturnal blood pressure and also decline circadian rhythm of BP [19]. In the second place, over activation of the rennin-angiotensin aldosterone system (RAAS) would produce more angiotensin II (AngII) that will increase cardiac afterload and affect circadian rhythm [20]. Furthermore, high-normal blood pressure could change the metabolism of blood lipids and blood glucose, and it might be a proinflammatory condition that changes the circadian rhythm [21].

According to our research, abnormal circadian rhythm and higher BPV are found in the high-normal blood pressure group, and some indicators are even more similar to hypertensive level. Those changes would be reasons that induce the target organ damage, increase the LVMI, and lead the left ventricular hypertrophy. So, it is meaningful to test the high-normal blood pressure group through ABPM and apply intervention treatment that could decrease the target organs damage and prevent hypertension.

## 4. Conclusion

After multiple relevant clinical confounding factors were adjusted, patients with dipper and nondipper high-normal blood pressure had higher LVMI. Abnormalities in circadian blood pressure variability may be associated with the left ventricular hypertrophy.

## Figures and Tables

**Table 1:** Baseline characteristics of the study population.

High-normal			Normotensive (*n* = 35)			
Dipper (*n* = 87)		Nondipper (*n* = 59)	*P*	*P*1	*P*1	*P*2
Age (years)	30 ± 6	31 ± 6	>0.05	31 ± 6	>0.05	>0.05
Family history	26	30	>0.05	12	>0.05	>0.05
BMI (kg/m^2^)	22.6 ± 2.8	22.6 ± 2.9	>0.05	20.9 ± 2.3	>0.05	>0.05
FBG (mmol/L)	0.90 ± 0.42	4.83 ± 0.83	>0.05	4.8 ± 0.29	>0.05	>0.05
TG (mmol/L)	1.0 ± 0.3	1.5 ± 0.8	>0.05	0.9 ± 0.4	>0.05	>0.05
TC (mmol/L)	4.5 ± 0.7	4.4 ± 0.6	>0.05	4.0 ± 0.6	>0.05	>0.05
HDL-C (mmol/L)	1.3 ± 0.3	1.1 ± 0.3	<0.05	1.3 ± 0.3	<0.05	<0.05
LDL-C (mmol/L)	3.0 ± 0.7	2.7 ± 0.6	<0.05	2.1 ± 0.6	<0.05	<0.05

*P*1 represents the *p* value of normotensive and dipper; *P*2 represents the *p* value of normotensive and nondipper. It is easier to find HDL-C and LDL-C have *p* < 0.05 which is considered as statistically significant.

**Table 2:** ABPM findings of high-normal and normotensive patients provided separately for dippers and nondippers.

High-normal			Normotensive (*n* = 35)			
Dipper (*n* = 87)		Nondipper (*n* = 59)	*P*	*P*1	*P*1	*P*2
24 h SBP (mmHg)	116.2 ± 10.6	118.8 ± 8.6	<0.05	101.2 ± 10.3	<0.05	<0.05
24 h DBP (mmHg)	76.9 ± 7.3	77.8 ± 6.4	<0.05	68.2 ± 6.6	<0.05	<0.05
DSBP (mmHg)	122.4 ± 11.0	120.3 ± 8.9	<0.05	103.4 ± 9.6	<0.05	<0.05
DDBP (mmHg)	81.1 ± 7.4	80.2 ± 6.6	<0.05	70.4 ± 7.0	<0.05	<0.05
NSBP (mmHg)	106.2 ± 10.6	113.4 ± 8.2	<0.05	99.2 ± 7.4	<0.05	<0.05
NDBP (mmHg)	67.8 ± 7.4	73.7 ± 6.7	<0.05	65.9 ± 5.1	<0.05	<0.05
24 h SBP CV (%)	10.7 ± 1.9	8.7 ± 1.5	>0.05	7.0 ± 1.0	>0.05	>0.05
24 h DBP CV (%)	13.7 ± 2.6	11.4 ± 3.1	>0.05	8.0 ± 1.2	>0.05	>0.05

*P*1 represents the *p* value of normotensive and dipper; *P*2 represents the *p* value of normotensive and nondipper. 24 h SBP, 24 h DBP, DSBP, DDBP, NSBP, and NDBP, *p* < 0.05 is considered significant.

**Table 3:** Correlation coefficient for the association between LVMI and other facts.

r		*t*/*P*
SBP	0.511	5.761/0.036
DBP	0.569	5.994/0.034
Patterns	0.726	7.761/0.028
Age	0.372	4.556/0.030
BMI	0.428	5.426/0.023
TG	0.589	4.761/0.017
TC	0.489	5.225/0.009
HDL-C	−0.452	6.437/0.010
LDL-C	0.533	4.263/0.021
24 h SBP CV	0.736	6.017/0.010
24 h DBPCV	0.699	5.343/0.008

## Data Availability

The data used to support the findings of this study are available from the corresponding author upon request.
